# The crystal structure of mycobacterial epoxide hydrolase A

**DOI:** 10.1038/s41598-020-73452-y

**Published:** 2020-10-06

**Authors:** Eike C. Schulz, Sara R. Henderson, Boris Illarionov, Thomas Crosskey, Stacey M. Southall, Boris Krichel, Charlotte Uetrecht, Markus Fischer, Matthias Wilmanns

**Affiliations:** 1 Max Planck Institute for the Structure and Dynamics of Matter, Luruper Chausee 149, 22761 Hamburg, Germany; 2 European Molecular Biology Laboratory, Hamburg Unit, Notkestrasse 85, 22603 Hamburg, Germany; 3 Hamburg School of Food Science, Institute of Food Chemistry, Universität Hamburg, Grindelallee 117, 20146 Hamburg, Germany; 4 Heinrich Pette Institute, Leibniz Institute for Experimental Virology, Martinistraße 52, 20251 Hamburg, Germany; 5 European XFEL GmbH, Holzkoppel 4, 22869 Schenefeld, Germany; 6 University of Hamburg Medical Center Hamburg-Eppendorf, Martinistraße 52, 20246 Hamburg, Germany; 7 Present Address: Norwich Medical School, Rosalind Franklin Road, Norwich Research Park, Norwich, Norfolk, NR4 7UQ UK; 8 Present Address: Sosei Heptares, Steinmetz Building, Granta Park, Great Abington, Cambridge, CB21 6DG UK

**Keywords:** Structural biology, X-ray crystallography

## Abstract

The human pathogen *Mycobacterium tuberculosis* is the causative agent of tuberculosis resulting in over 1 million fatalities every year, despite decades of research into the development of new anti-TB compounds. Unlike most other organisms *M. tuberculosis* has six putative genes for epoxide hydrolases (EH) of the α/β-hydrolase family with little known about their individual substrates, suggesting functional significance for these genes to the organism. Due to their role in detoxification, *M. tuberculosis* EH’s have been identified as potential drug targets. Here, we demonstrate epoxide hydrolase activity of *M. thermoresistibile* epoxide hydrolase A (Mth-EphA) and report its crystal structure in complex with the inhibitor 1,3-diphenylurea at 2.0 Å resolution. Mth-EphA displays high sequence similarity to its orthologue from *M. tuberculosis* and generally high structural similarity to α/β-hydrolase EHs. The structure of the inhibitor bound complex reveals the geometry of the catalytic residues and the conformation of the inhibitor. Comparison to other EHs from mycobacteria allows insight into the active site plasticity with respect to substrate specificity. We speculate that mycobacterial EHs may have a narrow substrate specificity providing a potential explanation for the genetic repertoire of epoxide hydrolase genes in *M. tuberculosis.*

## Introduction

Infectious diseases pose a major threat to societies in the twenty-first century as many bacteria have managed to evolve mechanisms that protect them from antibiotics^1^. Of particular concern is infection with *Mycobacterium tuberculosis* (*M. tuberculosis*) the causative agent of tuberculosis, which caused approximately 10.4 million cases in 2017, resulting in 1.3 million deaths in HIV-negative patients. Alarmingly, in excess of 4% of the cases in 2017 were found to be multi-drug resistant^2^. In addition to this, there have been several reports of totally drug resistant tuberculosis in recent years^3–5^. In spite of decades of intense research simple, effective solutions to treat tuberculosis are still unavailable^6^. These facts demonstrate that it is of paramount importance to gain a more comprehensive understanding of the biological foundations of *M. tuberculosis* to be able to treat tuberculosis in the future.

Substantial insight into *M. tuberculosis* biology was provided by sequencing of its complete genome, which revealed that a comparably large fraction of all genes codes for enzymes involved in lipogenesis and lipolysis^7,8^. While epoxide hydrolases (EHs) are found in about 20% of all organisms with sequenced genomes, most of them occur in organisms with large genomes (> 8 Mb). The mycobacterial genome harbors less than 4 Mb but contains on average five epoxide hydrolases compared to approximately 2 found in other organisms. Since evolutionary pressure drives bacteria to deleting unneeded genes, this comparably large number of EHs supports the idea of functional significance of these enzymes to *M. tuberculosis* biology^9^. Initially, six putative copies of epoxide hydrolases *ephA*–*ephF* (EHs; E.C. 3.3.2.3; open reading frames Rv3617, Rv1938, Rv1124, Rv2214c, Rv3670 and Rv0134) could be identified^7^. Later another open reading frame (Rv2740) was shown to produce an atypical epoxide hydrolase (E.C. 3.3.2.8)^10^. At present, four of these genes have been confirmed to produce proteins with resulting enzymatic activity of an epoxide hydrolase (*ephA*^this work^, *ephB*^11,12^, *ephD*^13^, *ephG*^10^). The idea to use mycobacterial EHs as potential drug targets was recently emphasized by screening of a large compound library in a whole cell phenotypic minimum inhibitory concentration (MIC) assay, which pointed towards EHs as the main target of the lead compound^14^.

EHs are essential enzymes that convert toxic epoxides to less reactive and more water soluble trans-dihydrodiols. They are generally implicated in detoxification of genotoxic epoxides but are also involved in more specialized processes like carbon catabolism and signal transduction. To date, three main classes of EHs have been identified; bacterial limonene EH (LEH), LTA_4_ hydrolases and α/β-hydrolase fold EH^15^. LEHs are essential for bacteria that use limonene as their only carbon source. The structural analyses of LEH from *Rhodococcus erythropolis* and an atypical EH from *M. tuberculosis* showed that these EHs do not belong to the family of α/β-hydrolases but rather consists of an α/β-barrel fold and follow a different reaction mechanism^10,16^. The second group EHs, the LTA_4_ hydrolases are Zn-dependent and show structural similarity to the bacterial protease thermolysin^17^. Similar to the bacterial LEHs, the LTA_4_ hydrolases display a specifically evolved active site and reaction mechanism^15^.

The majority of mycobacterial EHs have been predicted to be members of the α/β-hydrolase fold family, presenting the third and largest class of EHs. Significant insight into the reaction mechanism of this EH-family was gained by several crystal structures of bacterial and mammalian EHs^18–22^. The structures of the α/β-hydrolase family EHs can be divided into two subdomains; the catalytic ‘core-domain’ and the so-called ‘cap-domain’. The active site is located in a cleft between these two domains. The core domain contributes a conserved catalytic triad to the active site, while two strictly conserved tyrosine residues are located in the cap-domain. These tyrosine residues are suggested to affect epoxide polarization and facilitate ring opening^23,24^. The two-step reaction mechanism starts with an attack by the nucleophilic active site aspartate at one of the substrates epoxide carbons forming a covalent substrate-enzyme intermediate. Subsequently, a charge-relay pair between a general acid (aspartate/glutamate) and a general base histidine activates a water molecule. This leads to the hydrolysis of the covalent enzyme–substrate intermediate. A conserved histidine-glycine-X-proline (HGXP) motif that is generally found in α/β-hydrolases, together with the nitrogen atom of the residue following the nucleophile forms an oxyanion hole that is suggested to stabilize the intervening tetrahedral intermediate^23–27^. Computational evidence supports that this catalytic strategy is conserved for enzymes within this family^28^.

EHs of the α/β-hydrolase family are known to accept a broad spectrum of structurally diverse substrates. In mammals, the soluble EHs hydrolyze gem-di-, trans-di-, cis-di-, tri- and tetra-substituted epoxides, while the microsomal EHs hydrolyze mono- and cis- di-substituted epoxides. On a molecular level, this broad substrate spectrum could be explained by the *nucleophile elbow*, a sharp turn in the active site, which harbours the nucleophilic aspartate that is responsible for the initial substrate attack^11,15,18,23,24^. It has been suggested that an increased flexibility of this aspartate enables adaptation to the position of the substrate in the active site^15^. Due to the high structural similarity between mammalian and bacterial EHs, it would seem conceivable that mycobacterial α/β-hydrolase EHs show a similar versatility. However, the position of the nucleophilic aspartate and the other active site residues of *M. tuberculosis* epoxide hydrolase B (Mtb-EphB, Rv1938) remain unaltered in the inhibitor bound and ligand-free protein^11^.

In combination with the comparably large number of Ehs in mycobacteria this lets us speculate about an individual substrate selectivity, presumably targeting classes of different substrates^9^. In support of this hypothesis, the structure of Mtb-EphB has revealed a relatively small and hydrophobic active site and an altered substrate selectivity in comparison to mammalian or plant EHs^11^. However, our understanding of a particular active site geometry in relation to substrate specificity is limited by the available structural information, limited to Mtb-EphB, to date. Several EH inhibitors have not only shown antitubercular activity and low cytotoxicity but also to target several different EHs. However, these inhibitors have also targeted the human enzymes^14^. Therefore, in the design of epoxide hydrolase inhibitors for the treatment of TB, it is desirable to increase their specificity selecting only mycobacterial targets, which would be facilitated by structural knowledge of all mycobacterial EHs. From the six putative α/β-hydrolase EHs in *M. tuberculosis* only the structure of Mtb-EphB has been solved to date^11^. Although the crystallization of *M. tuberculosis* Epoxide hydrolase A (Mtb-EphA, Rv3617) has been reported, its limited X-ray diffraction prevented structure determination^12^. To enhance our structural understanding mycobacterial epoxide hydrolases, we have solved the crystal structure of the *M. thermoresistibile* Epoxide hydrolase A (Mth-EphA) in complex with a urea-based inhibitor at 2.0 Å resolution. The structure clearly displays an α/β-hydrolase EH fold but in comparison to other EHs shows substantial differences in the substrate binding channel.

## Results

### Protein characterization and enzyme activity assay

A sequence comparison of epoxide hydrolase *A* from *M. tuberculosis* (Mtb-EphA) and its temperature tolerant relative *Mycobacterium thermoresistibile* (Mth-EphA) shows that the two orthologs share approximately 75% sequence identity. All residues suspected to be involved in catalysis are identical (Figure S1). This demonstrates that Mth-EphA may serve well as a model to understand the molecular details of Mtb-EphA. Consequently, the gene for Mth-EphA was cloned and expressed in *E. coli*, the recombinant protein was purified to homogeneity.

**Figure 1:**
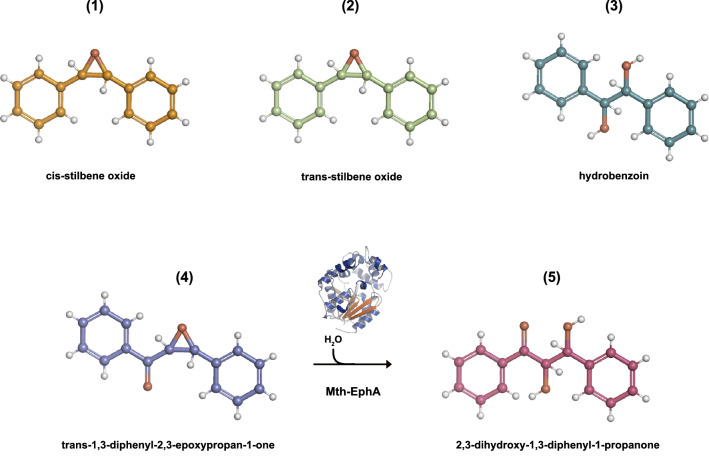
Mth-EphA has epoxide hydrolase activity. 2D-projections of compounds (**1**)–(**5**): cis-stilbene oxide (**1**), trans-stilbene oxide (**2**), hydrobenzoin (**3**), trans-1,3,diphenyl-2,3-epoxypropan-1-one (**4**) and 2,3dihydroxy-1,3diphenyl-1-propanone (**5**). LC–MS based activity assays demonstrate that Mth-EphA converts (**4**) into (**5**) but not (**1**) or (**2**) into (**3**).

To qualitatively determine whether Mth-EphA has indeed epoxide hydrolase activity we incubated the protein with following compounds: cis-stilbene oxide (**1**), trans-stilbene oxide (**2**), or trans-1,3-diphenyl-2,3-epoxypropan-1-one (**4**) (Fig. 1) and analyzed its reaction products by liquid chromatography mass-spectrometry (LC–MS) (Table 1). Treatment of **1** or **2** with Mth-EphA did not reduce the concentration of any of those two compounds in the assay samples and did not give rise to hydrobenzoin (**3**) (Fig. S5–S7). However, treatment of **4** with Mth-EphA reduced the concentration of its adducts (H^+^, NH_4_^+^ and Na^+^, retention time 37.5 min) in the measured samples approximately to half of the initial values and gave rise to three signals with m/z values of 243Th, 260Th and 265Th, all with identical retention time of 15 min. We concluded that these molecular species must be three adducts (H^+^, NH_4_^+^ and Na^+^, respectively) of the chemical compound with molecular weight of 242 Da, which is characteristic to the hydroxylation product of **4**, 2,3-dihydroxy-1,3-diphenyl-1-propanone (**5**) (Table 1 and Fig. S8–S11). The latter is commercially not available. Therefore, for fragmentation of those three molecular species (**5**-H^+^, **5**-Na^+^, **5**-NH_4_^+^) and the three adducts of **4** (**4**-H^+^, **4-**Na^+^, **4-**NH_4_^+^) the MRM parameters established for **4** have been used. The species **4**-Na^+^ and **5**-Na^+^ as expected did not give any measurable fragments because sodium adducts normally tend to produce very low fragmentation. The molecular species **4**-H^+^, **4-**NH_4_^+^, **5**-H^+^ and **5-**NH_4_^+^, all produced three fragments with m/z of 51Th, 77Th and 105Th (cf. Table 1, fragments **4**-1, **4**-2, **4-**3, **5-**1, **5-**2, **5-**3). While the first is characteristic for fragmentation of phenols, the second and third correspond to fragmentation results of substituted phenols. For the molecular species **5**-H^+^ and **5**-NH_4_^+^ fragments with m/z of 119Th and 225Th could be observed. The first species can be easily identified as 2-oxo-2-phenylethylium (**5-**4), while the second species (**5-**5) has an *m/z* value identical with **4**-H^+^. In other words **5** showed neutral loss of water which is a typical fragmentation pattern of alcohols. A loss of a second molecule of water by fragmentation of the **5-**5 would be conceivable if the **5-**5 was an alcohol, too. But the putative product of such fragmentation with *m/z* 197Th (C_15_H_11_O_1_^+^) was not observed. Thus, we concluded that under neutral loss of one water molecule the diol (**5**) is converted to a dicarbonyl compound (**5**-5) in a way similar to the pinacol rearrangement. Therefore the **5-**5 must be a protonated 1,3-diphenylpropanedione and not 1,3-diphenylhydroxypropanone.

**Table 1:** Adducts of compounds cis-stilbene oxide (**1**), trans-stilbene oxide (**2**), hydrobenzoin (**3**), trans-1,3-diphenyl-2,3-epoxypropan-1-one (**4**), 2,3-dihydroxy-1,3-diphenyl-1-propanone (**5**), the MRM parameters optimized for their fragmentation and the resulting fragmentation products.

Compound^a^	Retention time, min	Adduct or fragment	*m/z* (Th)	Chemical formula	Chemical name of the compound/fragment ion	CE, eV	CXP, V
**1**	32	**1**-H^+^	197	C_14_H_13_O^+^	Cis-stilbene epoxide, H^+^ adduct	5	10
		**1**-Na^+^	219	C_14_H_12_ONa^+^	Cis-stilbene epoxide, Na^+^ adduct	5	10
		**1**-NH_4_^+^	215	C_14_H_16_ON^+^	Cis-stilbene epoxide, NH_4_^+^ adduct	5	10
		**1**-1	51	C_4_H_3_^+^	Cyclobutadienylium	91	0
		**1**-2	77	C_6_H_5_^+^	Phenylium	49	4
		**1**-3	91	C_7_H_6_^+^	Tropylium	19	6
		**1**-4	105	C_7_H_5_O^+^ or C_8_H_9_^+^	Oxo(phenyl)methylium or phenylethylium	25	6
**2**	32	**2**-H^+^	197	C_14_H_13_O^+^	Trans-stilbene epoxide, H^+^ adduct	5	10
		**2**-Na^+^	219	C_14_H_12_ONa^+^	Trans-stilbene epoxide, Na^+^ adduct	5	10
		**2**-NH_4_^+^	215	C_14_H_16_ON^+^	Trans-stilbene epoxide, NH_4_^+^ adduct	5	10
		**2-**1	51	C_4_H_3_^+^	Cyclobutadienylium	85	14
		**2**-2	77	C_6_H_5_^+^	Phenylium	51	12
		**2**-3	91	C_7_H_6_^+^	Tropylium	19	6
		**2**-4	105	C_7_H_5_O^+^ or C_8_H_9_^+^	Oxo(phenyl)methylium or phenylethylium	29	6
**3**	13	**3**-H^+^	215	C_14_H_15_O_2_^+^	Hydrobenzoin, H^+^ adduct	5	10
		**3**-Na^+^	237	C_14_H_14_O_2_Na^+^	Hydrobenzoin, Na^+^ adduct	5	10
		**3**-NH_4_^+^	232	C_14_H_18_O_2_N^+^	Hydrobenzoin, NH_4_^+^ adduct	5	10
		**3**-1	51	C_4_H_3_^+^	Cyclobutadienylium	93	8
		**3**-2	77	C_6_H_5_^+^	Phenylium	57	6
		**3**-3	91	C_7_H_6_^+^	Tropylium	25	6
		**3**-4	105	C_7_H_5_O^+^ or C_8_H_9_^+^	Oxo(phenyl)methylium or phenylethylium	27	5
**4**	37.5	**4**-H^+^	225	C_15_H_13_O_2_^+^	1,3-diphenyl-2,3-epoxy-1-propanone, H^+^ adduct	5	10
		**4**-Na^+^	247	C_14_H_12_O_3_Na^+^	1,3-diphenyl-2,3-epoxy-1-propanone, Na^+^ adduct	5	10
		**4**-NH_4_^+^	242	C_14_H_16_O_3_N^+^	1,3-diphenyl-2,3-epoxy-1-propanone, NH_4_^+^ adduct	5	10
		**4**-1	51	C_4_H_3_^+^	Cyclobutadienylium	89	6
		**4**-2	77	C_6_H_5_^+^	Phenylium	49	4
		**4**-3	105	C_7_H_5_O^+^	Oxo(phenyl)methylium or phenylethylium	13	8
**5**	15.0	**5**-H^+^	243	C_15_H_15_O_3_^+^	2,3-dihydroxy-1,3-diphenyl-1-propanone, H^+^ adduct	5	10
		**5**-Na^+^	265	C_15_H_14_O_3_Na^+^	2,3-dihydroxy-1,3-diphenyl-1-propanone, Na^+^ adduct	5	10
		**5**-NH_4_^+^	260	C_15_H_17_O_3_N^+^	2,3-dihydroxy-1,3-diphenyl-1-propanone, NH_4_^+^ adduct	5	10
		**5**-1	51	C_4_H_3_^+^	Cyclobutadienylium	89	6
		**5**-2	77	C_6_H_5_^+^	Phenylium	49	4
		**5**-3	105	C_7_H_5_O^+^ or C_8_H_9_^+^	Oxo(phenyl)methylium or phenylethylium	13	8
		**5**-4	119	C_8_H_7_O^+^	2-Oxo-2-phenylethylium	11	10
		**5**-5	225	C_15_H_13_O_2_^+^	1,3-diphenylpropanedione, H^+^ adduct	5	10

*CE* collision energy, *CXP* collision cell exit potential.

^a^Declustering potential values (V): 56 (**1**), 66 (**2**), 44 (**3**), 31 (**4**), 31 (**5**).

In conclusion, the LC–MS data are consistent with **5** being 2,3-dihydroxy-1,3-diphenyl-1-propanone indicating that Mth-EphA can convert **4** to **5** under described reaction conditions. By contrast, the hydroxylation of the two other potential EH substrates **1** and **2** was not observed.

### Structure determination

The purified protein was used in crystallization experiments which following optimization, yielded crystals diffracting to 2.0 Å resolution (Table 2, Figure S1). The structure was solved by molecular replacement using a homology model as template. Despite the challenging diffraction properties of the crystals, the data yielded an electron-density map with clearly interpretable features allowing accurate model building for residues 4–321 of the protein construct. All residues lie within allowed Ramachandran areas, with the exception of the active site residue Asp103. The strained conformation of Asp103 can likely be explained by its position at the tip of the nucleophilic elbow. Notably, Asp103 displays very well-defined electron density and its unfavorable conformation is held in position by interactions to the surrounding residues. Asp103 forms backbone hydrogen bonds to Ala106, Leu107 as well as Leu126 and its side chain carboxyl group contacts the backbone nitrogens of Phe35 and Trp104. Although four monomers were observed in the asymmetric unit, no significant dimer interface that would lead to the formation of a stable quaternary structure was determined in PISA analysis^29^.

**Table 2:** Data collection and refinement statistics.

Data collection		Refinement	
Wavelength (Å)	0.9752	Resolution limits (Å)	47.82–2.0
Cell dimensions (Å)		No. reflections	93,380
a	61.80	No. atoms	10,371
b	105.01	Macromolecules	9898
c	108.95	Ligands	64
α	90	Water	408
β	90	R_Work_ (%)/R_Free_ (%)	20.86/25.08
γ	90		
Space group	P 1 2_1_ 1	B-factors	
Resolution range (Å)	47.82 -2.0	Macromolecules	22.00
No. reflections	316,649 (32,211)	Solvent	21.30
I/σ	7.68 (2.42)	Ligands	18.80
Completeness (%)	96.7(97.5)	R.m.s. deviations	
Redundancy	3.4 (3.5)	Bond lengths (Å)	0.014
R_Meas_ (%)	13.63	Bond angles (°)	1.5
CC(1/2)	0.994 (0.83)		

This is consistent with native mass spectrometry (MS) analysis demonstrating that Mth-EphA is a monomer in solution (Figure S1). When sprayed from a nano-ESI (electrospray ionization) source the peaks appeared in a narrow charge state distribution with mainly + 10 and + 11 charges indicating a well-defined fold. A dissociation constant (K_D_) can be estimated from the relative peak intensities of apo Mth-EphA (100%) compared to Mth-EphA bound to 1,3-diphenylurea (DPU) (5%). However, the intensity ratios will not reflect the K_D_ of such a hydrophobic interaction. Generally, non-polar interactions are poorly preserved in the gas phase and unspecific clustering rarely occurs. Detecting this interaction suggests that it is in fact specific, and that it would likely be much stronger in solution. Moreover, such a small hydrophobic ligand would be unlikely to detect at all if not buried inside the molecule^30^.

### Overall structure

**Figure 2:**
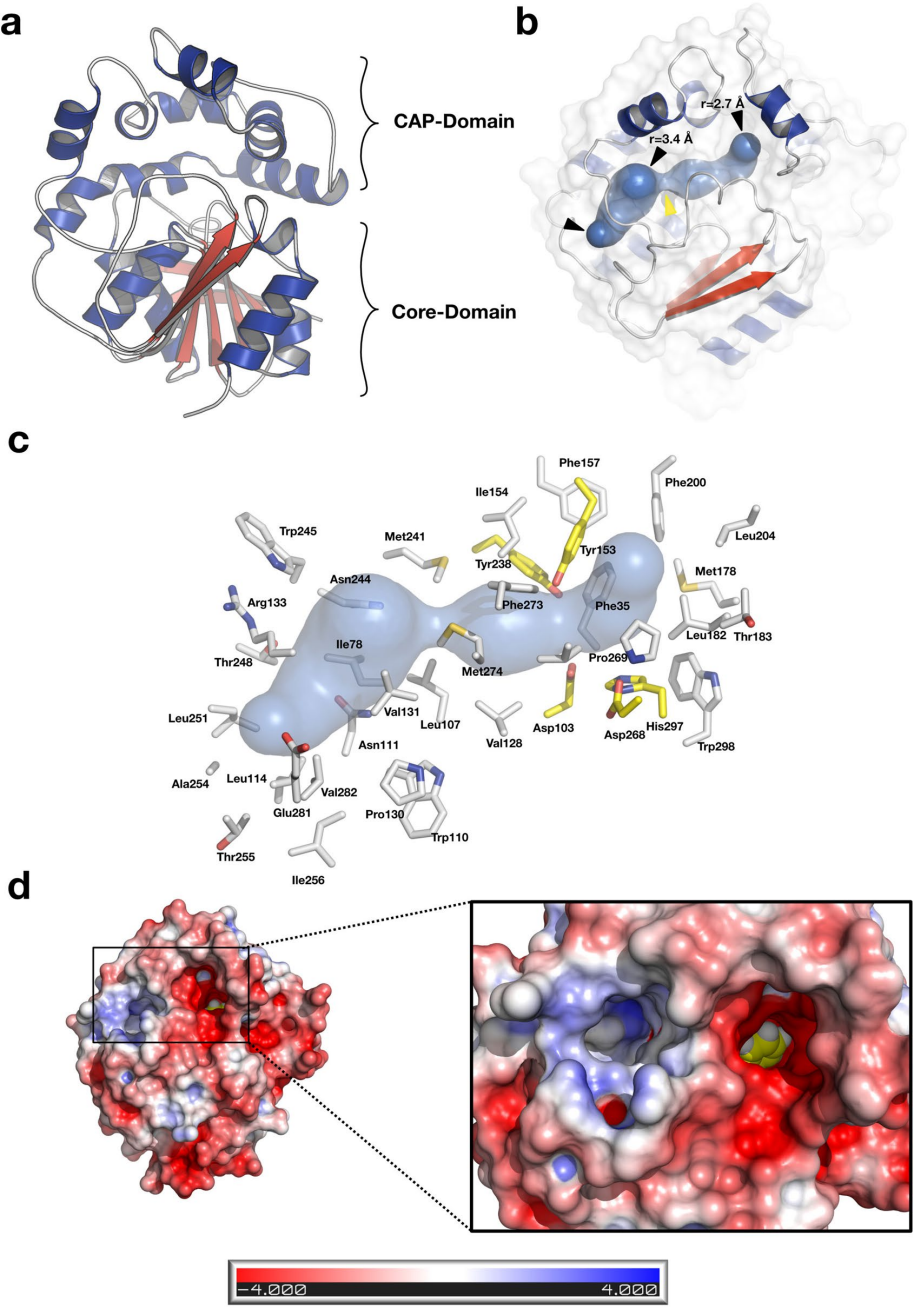
Structural overview of Mth-EphA. Cartoon ribbon representation of Mth-EphA, α-helices are colored in blue, β-strands are colored in red, loops are in grey. (**A**) Mth-EphA belongs to the α/β-hydrolase fold family of enzymes. The α/β-hydrolase domain features its characteristic central β-sheet with α-helices aligned to its sides. Clearly the structure is organized into two separated domains: the all-α cap domain closing over the α/β-hydrolase fold domain. (**B**) The course of the substrate tunnel is indicated as a blue sphere inside of Mth-EphA; black arrowheads indicate solvent accessible openings; a yellow arrowhead marks the position of the constriction site. (**C**) Side chains lining the tunnel are indicated as grey stick, catalytic residues are shown in yellow. (**D**) APBS surface charge representation of Mth-EphA. The surface charges clearly show the acidic (red) and alkaline (blue) charges on the surface of Mth-EphA. While the cap-domain is predominantly acidic the core-domain has less pronounced charges. Particularly interesting are the entry sites to the substrate channel, which display opposite charges on the surface of the protein. It is conceivable that these features are involved in substrate orientation or selection. The inhibitor in the active site is visible as yellow spheres.

The overall structure of Mth-EphA clearly displays the globular fold of the α/β-hydrolase EHs. It is separated into two domains; the α/β-hydrolase core-domain (residues 1–136 and 247–321) with its central, eight-stranded β–sheet and the all-α-helical cap-domain (residues 137–246) (Fig. 2). The interface between cap-domain and core-domain harbors the active site of Mth-EphA, with the conserved residues of the catalytic triad (Asp103, His297 and Asp268) and the epoxide polarizing tyrosines (Tyr153, Tyr238) (Fig. 2). The cap-domain closes over the core-domain leaving space for a characteristic ‘L’-shaped tunnel with a hydrophobic interior (Fig. 2). The tunnel has an overall length of approximately 30 Å and bulky cavities close to its two distinct solvent openings, with radii of 2.7 Å and 3.4 Å, respectively (Figure S2). However, at its center, in proximity to the active site residues, it shows a constriction with a radius of only 1.2 Å. This constriction is imposed by three hydrophobic residues (Leu107, Met241, Met274) and presumably influences the substrate selection by Mth-EphA. A surface charge calculation shows a generally acidic surface for the cap-domain of Mth-EphA, while the surface charges at the core-domain are less pronounced. Interestingly, the two openings to the substrate-channel display opposite charges. While one opening has a strongly acidic surrounding the other shows clear alkaline properties (Fig. 2). It is conceivable that these properties influence substrate selection or guide substrate orientation and binding.

### Inhibitor binding

**Figure 3:**
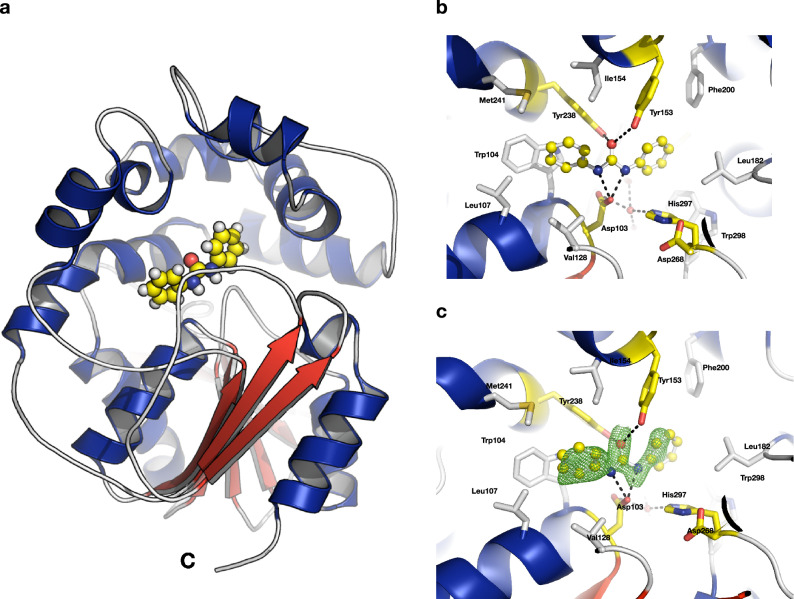
Inhibitor binding. (**A**) The 1,3-diphenylurea ligand is indicated by yellow spheres in the active site between the cap-domain and the α/β-hydrolase domain. (**B**) The inhibitor 1,3-diphenylurea binds in the active site residues of Mth-EphA. Catalytic residues are shown in yellow. (**C**) A simulated annealing OMIT map of the inhibitor (|Fo-Fc|, shown at 2 σ), is displayed as a green mesh.

The previously established EH inhibitor 1,3-diphenylurea was co-crystallized with Mth-EphA. The inhibitor is deeply buried within the substrate tunnel, where it binds to the conserved active site residues (Fig. 3). The conformation of the inhibitor binding could be unambiguously determined from the electron density maps. While both of the amine nitrogens form hydrogen bonds with Asp103, the phenyl groups are stabilized by van der Waals interactions (Phe35, Met178, Leu182, Phe200, Trp298 and Trp104, Leu107, Ile154, Met241). The carbonyl oxygen of the inhibitor is hydrogen bonded by Tyr153 and Tyr238 mimicking the position of the epoxide oxygen. Interestingly, the inhibitor adopts a slightly bent conformation with the carbonyl group and both phenyl-rings oriented towards Tyr238. In proximity of Asp103 and His297 a water molecule shows electron density and is stabilized by hydrogen bonds to the side chains of those residues, to Glu39 as well as to the main chain of Phe35 (Fig. 3).

## Discussion

**Figure 4:**
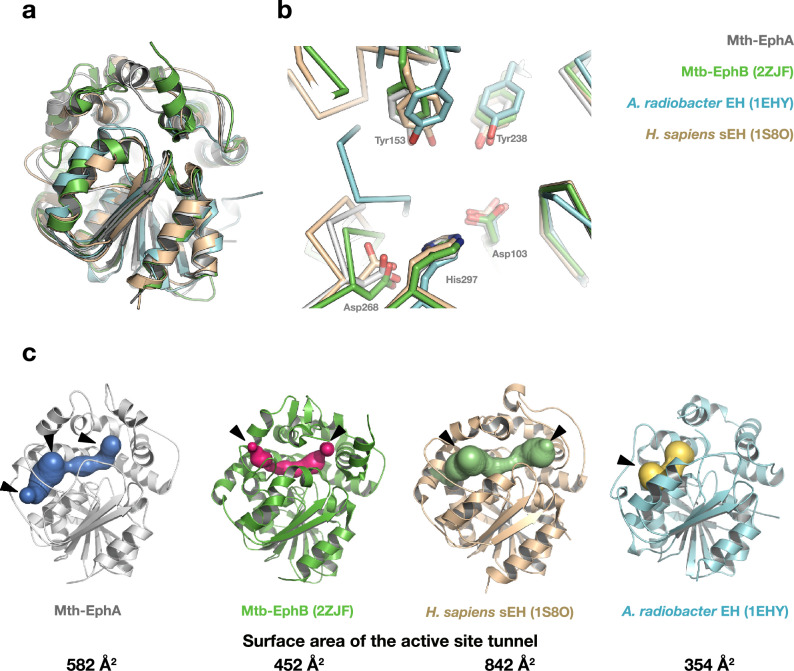
Comparison to other α/β-hydrolase EHs. EHs display a high degree of structural conservation, that is not only limited to their main-chain atoms but can also be seen on the side chain level in the active site. However, substantial differences can be observed in the size, shape and propagation of the substrate channels. (**A**) A superposition of Mth-EphA with Mtb-EphB, human sEH and the EH from *A. radiobacter* displays their global similarity. (**B**) A close-up to the catalytic residues of the superposed EHs illustrates the structural conservation in the active site. (**C**) In spite of the global similarity of the EHs their substrate channels clearly adopt different sizes, shapes and propagate into different directions. For clarity the N-terminal residues of human sHE are not shown. The surface area of the substrate channels is shown below the structures.

*M. thermoresistibile* epoxide hydrolase A has high sequence similarity to its orthologue from *M. tuberculosis* and its crystal structure described here displays overall high structural similarity to α/β-hydrolase EHs. To gain insight into its substrate binding properties and shed light on its potential substrate specificity, we compare its substrate channel with that of another bacterial EH and the human orthologue. The structure of Mth-EphA presented here can be superimposed onto the structures of Mtb-EphB (2ZJF), to the EH from *Agrobacterium radiobacter* (1EHY) and to the catalytic domain of human soluble epoxide hydrolase (sEH; 1S8O) with an average r.m.s.d. of less than 0.5 Å. Moreover, the global similarity of the EHs is not limited to their main chains but also extends to the catalytic residues in the active site, which directly overlap upon superposition (Fig. 4).

However, in spite of the conserved active site conformation, substantial differences in the substrate channels can be observed. Mth-EphA features a wide double cavity that is connected by a narrow constriction at its center (Figs. 2, 4, Figure S2 and S3). The channel has two major openings to the solvent that exhibit opposite surface charges, while the other EHs compared here do not feature opposite charges at their active site entry sites. Mtb-EphB, which shares 40% sequence identity with Mth-EphA, on the other hand has a much narrower substrate channel with only two minor connections to the solvent. Moreover, upon superposition of the structures it is evident that the individual substrate channels not only have different shapes and sizes but also propagate along different directions.

Similar to the mycobacterial EHs, the human sEH channel also has two openings to the solvent. However, clearly distinct from the mycobacterial EHs, the channel of the human orthologue appears to be wide enough to accept a variety of different and bulky substrates. This is consistent with its general versatility accepting differentially substituted epoxy fatty acids as substrates^16,23,31^. In contrast to the former structures, the EH from *A. radiobacter* has only one large opening on its surface and a shallow connection to the active site that is not fully shielded from the solvent (Fig. 4, Figure S4). It appears to be better suited to accommodate smaller substrates with epoxides on terminal positions consistent with its primary, natural substrate epichlorohydrin^32,33^.

**Figure 5:**
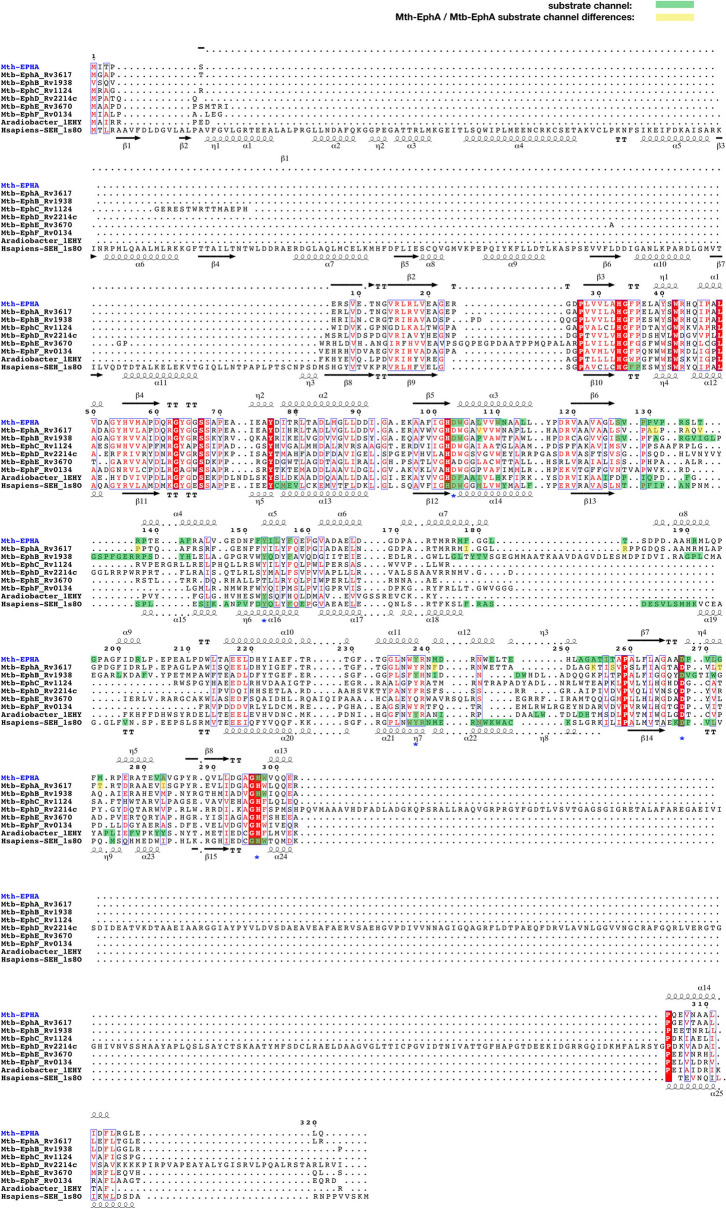
Sequence alignments of mycobacterial EHs. Sequence and secondary structure alignment of Mth-EphA, the six putative *M. tuberculosis* α/β-hydrolase family EHs, *A. radiobacter* EH and human soluble EH. Secondary structure annotations are derived from Mth-EphA and human sHE. Substrate channel lining residues are colored in green, those that deviate between Mth-EphA and Mtb-EphA are highlighted in yellow, catalytic residues are indicated with a blue asterisk. Clearly the substrate channel lining residues (green) map to the variable regions in the α-helical cap-domain, between strands β6 and β7. Most of the substrate channel lining residues are identical in Mth-EphA and Mtb-EphA, however, species–specific mutation can be observed for ~ 27% of the residues.

Unfortunately, to the best of our knowledge the actual substrate specificity of mycobacterial EHs are unknown. However, a comparison of the affinities of inhibitors for human sHE and Mtb-EphB indicates substantial differences between the two enzymes. Both of them can bind smaller inhibitors like 1,3-diphenylurea with high affinity. In consistence with its narrow substrate channels, Mtb-EphB shows much lower affinity to those inhibitors that exhibit long or bulky side-chains^11^. This suggests that its natural substrates could also be smaller molecules. The substrate channel of Mth-EphA is clearly distinct from the one in Mtb-EphB, showing two bulky cavities, a constriction at its center and highly polar entry sites (Fig. 2). It is conceivable that these features could play a role in substrate orientation and selection. In line with these findings Mtb-EphB shows activity against both cis- and trans-stilbene oxide while Mth-EphA did not show any activity against either of the compounds in our assay^11^. In summary, the properties of the active site channels as well as the results of our activity assay support the view that the comparably large genetic repertoire in mycobacterial epoxide hydrolases may be related to the substrate specificity of the individual enzymes. In line with this a sequence alignment reveals that the residues that line the substrate channel predominantly map to the most variable parts of the EHs, that is the α-helical cap-domain (Fig. 5). Similar to Mth-EphA and Mtb-EphB, the remaining mycobacterial EHs also show substantial sequence variability in their putative substrate channel locations. This further supports our view that mycobacterial epoxide hydrolases may have varying substrate specificities. Thus, we speculate that the large genetic repertoire of EHs in *M. tuberculosis* may have evolved to address different substrate classes.

The nature of the substrate of EHs is likely to have important kinetic consequences: human soluble EHs are able to process a large number of structurally diverse substrates following a 2-step reaction mechanism whereas the LEH from *Rhodococcus erythropolis* has a very narrow substrate specificity and enantio-selectivity. However, this narrow specificity is linked to a high turnover number of its main substrate maintaining its efficient hydrolysis^15,16,34,35^. By analogy, it is conceivable that a narrow substrate specificity of mycobacterial EHs could be linked to higher turnover rates. Therefore, the evolution of a large number of mycobacterial EHs could be an adaptation for the efficient hydrolysis of different substrate classes.

This is in good agreement with other physiological properties of *M. tuberculosis*. Since a large fraction of its genome is dedicated to the lipid metabolism it is reasonable to assume that it also requires an increased number of lipid-modifying enzymes. Moreover, the degradation of host cell lipids is vital to *M. tuberculosis,* as they serve as precursors for metabolic processes and are utilized as components of the mycobacterial cell wall^7^. This hypothesis gains support from recent findings that show up-regulated expression of epoxygenases in human macrophages, the natural habitat of *M. tuberculosis*^36,37^*.* Thus, it is conceivable that due to its genetic repertoire in EHs, *M. tuberculosis* is able to exploit the modified fatty acids present in human macrophages. Moreover, epoxygenases were also recently implicated in infection with intracellular bacteria underlining the potential importance for mycobacterial EHs during infection^38^.

Clearly, further research is required to fully understand the importance of EHs and the role they play during mycobacterial infections. Their overall structure is highly similar to the human soluble EH rendering an inhibitor design that targets the catalytic residues a challenging task. However, the structure of Mth-EphA described here furthers our understanding of the mechanism by which EHs might achieve substrate specificity and points to new opportunities for modulation of their activity. Further crystal structures of the remaining EHs (EphC-F) would add to our understanding of the nuances of mycobacterial EHs and facilitate design of EH-inhibitors that target other features of the proteins. The subtype specific substrate channels appear to be an interesting goal for future studies—not only limited to inhibitor design but also to better understand mycobacterial EH substrate specificity and thus their interaction with the host cell.

## Methods

### Protein purification

The gene encoding *Mycobacterium thermoresistibile* epoxide hydrolase A (Mth-EphA) (gi 490022613) was cloned into the pETM-11 vector (EMBL); protein expression was conducted in *Escherichia coli* Rosetta 2 (DE3) in LB medium at 20 °C for 18 h. The Mth-EphA construct used in this study contained all amino acids of the 321-residue protein. At the N-terminus it contained 4 additional residues (Gly-Ala-Met-Ala) linked to a TEV protease cleavage site and His_6_-tag. The His_6_-fusion protein was purified via affinity chromatography on a Ni–NTA resin (Quiagen) following the manufacturer’s instructions (50 mM Tris/HCl pH 7.5, 300 mM NaCl, 5% (v/v) glycerol). The His tag was removed by addition of TEV protease and dialysis at 4 °C for 18 h (50 mM Tris/HCl pH 7.5, 50 mM NaCl, 5% (v/v) glycerol). To remove the cleaved His_6_-tag and the TEV protease the solution was further purified via a MonoQ 10/100 ion exchange column (50 mM Tris/HCl pH 7.5, 5% (v/v) glycerol, 50–300 mM NaCl). EphA was further purified by Superdex S-75 size exclusion chromatography (50 mM Tris/HCl pH 7.5, 150 mM NaCl). Protein purity is not only reflected in a sharp, mono-dispersed elution profile in size exclusion chromatography but also in clear bands on SDS–polyacrylamide gels (Figure S1). Purified EphA was concentrated to 18 mg/ml and stored at − 80°°C until further use.

### Native mass spectrometry (MS)

Purified Mth-EphA was exchanged into 150 mM ammonium acetate (AmAc) (PN431311, 99.99% purity, Sigma-Aldrich, MO, USA), pH 7.5, via centrifugal filter units (Vivaspin 500, MWCO 10,000, Sartorius, Germany) at 4 °C. The near physiological conditions allow on one hand preservation of the native fold of the protein and on the other hand prevent adducts by nano-ESI. For binding experiments a 500 mM DPU stock in DMSO was diluted 50:1 in 150 mM AmAc in order to produce a saturated solution, considering the maximum DPU solubility in aqueous solutions of 710 µM/L (The Merck Index. 9th ed. Rahway, New Jersey: Merck & Co., Inc., 1976., p. 227) with and without 10% (v/v) butanediol, since it was also used for crystallization. Prior to measurements the inhibitor mix was diluted and incubated with EphA, resulting in 6 μM EphA, 71 μM DPU and 0.2% DMSO. Native MS was carried out in positive ion mode on a QToF 2 (Micromass/Waters, UK) modified for high masses (MS Vision, the Netherlands)^39^. The gas pressures were 10 mbar in the source region and 1.2 × 10^–2^ mbar Argon in the collision cell. Raw data were calibrated with CsI (25 mg/mL) and analyzed using MassLynx (Waters). Peak deconvolution and determination of relative intensity was performed using UniDec^40^.

### LC–MS based enzyme activity assay

Stock solutions of cis-stilbene oxide (**1**), trans-stilbene oxide (**2**), hydrobenzoin (1,2-diphenyl-1,2-ethandiol) (**3**), trans-1,3-diphenyl-2,3-epoxypropane-1-one (**4**) were prepared in methanol LC–MS grade (each 20 mM) and kept at − 80 °C. Assay and control samples were prepared as follows: To 200 µl of the assay buffer (25 mM Tris-formic acid pH 7.2) 4 µl of one of the potential EH substrates (**1**, **2** or **4**, final concentration in the assay 400 µM) was added. The reaction in the assay samples was started by addition of 0.5 µl of 20 µM enzyme solution (final concentration 0.025 µM). To control samples no enzyme was added. The assay and control a samples were incubated 60 min at 30 °C. Thereafter the samples were chilled on ice for 3 min and the reaction was quenched by addition of 800 µl of ice-cold methanol. After incubation on ice for 10 min the samples were centrifuged 30 min, 13,000 rpm, 0 °C. The supernatant was collected and stored at − 80 °C. The supernatant samples were analyzed on HPLC1200 (Agilent) equipped with PRP C18 column (150 mm × 2.1 mm, Hamilton) and coupled to API4000 Q-Trap Triple Quad mass spectrometer (SCIEX). Mobile phase A was 3 mM ammonium formate in water:methanol (1:1, v/v), mobile phase B was 3 mM ammonium formate in water:methanol (1:995, v/v). Aliquots (20 µl) were injected onto the column equilibrated with the mobile phase A. The column was developed with a gradient of the mobile phase A: 100%, at 0 min; 0% at 10 min; 0% at 45 min; 100%, at 46 min; 100%, at 70 min. The flow rate was 200 µL/min. MRM transition parameters (electrospray ionization, positive mode) for four available standard compounds were optimized using the Compound Optimization tool of the software Analyst (SCIEX). The *m/z* values, transition parameters and chemical formula of five compounds as well as their most populated fragmentation products are shown in the Table 1. The MRM chromatograms are shown in the supplemental material (Fig. S5–S11).

### Protein crystallization

Mth-EphA displayed high sensitivity towards DMSO. Thus, an inhibitor complex was generated by diluting a 1,3-diphenylurea solution (100 mM in 100% DMSO) 1:50 (v/v) with the protein solution, the resulting precipitate was removed by centrifugation, the supernatant was used for crystallization trials. Mth-EphA crystallized in multiple conditions, however, best diffracting crystals were only obtained in presence of low concentrations of 2,3-butanediol (1–3% v/v). Crystals were grown at 18 °C in sitting drop vapour diffusion plates combining equal volumes of precipitant (0.1 M BisTris pH 7.5; 2.15 M (NH_4_)_2_SO_4_; 4.5% (v/v) PEG 400; 1–3% (v/v) 2,3-butanediol) and the protein solution. The crystals appeared infrequently, as thin and often coadunate plates within 2–3 weeks and grew to a size of approximately 100 × 80 × 10 µm (Fig. S1). Crystals were cryo-protected by soaking in precipitant solution containing additionally 12% (v/v) 2,3-butanediol. Crystals were flash frozen in liquid nitrogen prior to data collection.

### Structure determination

X-ray data collection was performed at 100 K; diffraction images were collected at beamline ID30A-2 at ESRF, Grenoble, using the automated MASSIF data collection program^41,42^. Diffraction images were indexed, integrated and scaled using XDS and XSCALE, respectively^43,44^. Initial indexing of the diffraction patterns indicated an orthorhombic space group. However, analysis with PHENIX.XTRIAGE revealed a strong off-origin Patterson peak of ~ 56% origin intensity indicating pseudo-translational symmetry^45^. Moreover, it also revealed that the data are twinned by pseudo-merohedry (α ~ 33%; twin law h, -k, -l), which was later accounted for during structure refinement. These features suggested a lower symmetry space group, which was confirmed by an analysis with ZANUDA^46^. The crystals obtained in this study belonged to space group P2_1_ (a = 61.8, b = 105, c = 108.9; α, β, γ = 90°) with four monomers in the asymmetric unit and diffracted up to a resolution of 2 Å. The structure was solved by molecular replacement (PHASER) using a homology model generated with the PHYRE2 server^47,48^. Model building was performed in COOT^49^. Coordinates were refined to reasonable stereochemistry using PHENIX.REFINE (Table 2)^45^. Alternating steps of refinement and structure adjustments were performed until the R-values converged. The structure was validated using MOLPROBITY^50^. It was refined to R_work_ and R_free_ values of 20.86% and 25.08%, respectively (Table 2). All Mth-EphA monomers can be superimposed to each other with an r.m.s.d. of less than 0.3 Å, demonstrating their structural equivalence. Refined coordinates were deposited in the RCSB as entry **5CW2.**

### Structure and sequence comparison

Protein sequences were derived from Tuberculist and aligned using TCOFFEE^51,52^. Sequence alignments were subsequently visualized using ESPRIPT2^53^. Structure analysis was conducted using tools from the CCP4 suite as well as using software from the PHENIX package^45,54^. Substrate tunnels were calculated using the CAVER3.0 plugin to PYMOL. After superimposition of the individual structures the carbonyl oxygen from the 1,3-diphenylurea inhibitor was used as a common starting point, using default values for optimization. The probe radius was set to 1, the shell depth to 4 and the number of approximating balls to 20 for all protein structures. The shell radius and the clustering threshold were chosen individually to retrieve the most prominent tunnels (shell radius/clustering threshold: 1S8O-4/7; 1EHY-4/3.5; 2ZJF-3/3.5; Mth-EphA-3/3.5)^55^. Overlapping tunnels have been merged for visualization.

### Molecular visualization

All molecular images were generated in PYMOL (Schrödinger LLC).

## Supplementary information

Supplementary Figures.

## Data Availability

Coordinates and structure factors have been deposited in the Protein Data Bank under accession codes 5CW2.
