# Supplementary material for: The crystal structure of mycobacterial epoxide hydrolase A
Source: Sci Rep. 2020 Oct 6;10:16539. doi: 10.1038/s41598-020-73452-y (PMC7538969; doi:10.1038/s41598-020-73452-y)
Supplement: Supplementary file 1 — Supplementary Figures. [file 41598_2020_73452_MOESM1_ESM.pdf]

The crystal structure of mycobacterial Epoxide Hydrolase A

Eike C. Schulz[1,2,*], Sara R. Henderson[2,†], Boris Illarionov[3], Thomas Crosskey[2], Stacey M. Southall[2,¿], Boris Krichel[4], Charlotte Uetrecht[4,5], Markus Fischer[3] and Matthias Wilmanns[2,6]

[1]Max Planck Institute for the Structure and Dynamics of Matter, Luruper Chausee 149, 22761 Hamburg, Germany;

[2]European Molecular Biology Laboratory, Hamburg Unit, Notkestrasse 85, 22603, Hamburg, Germany;

[3]Hamburg School of Food Science, Institute of Food Chemistry, Universität Hamburg, Grindelallee 117, D-20146 Hamburg, Germany

[4]Heinrich Pette Institute, Leibniz Institute for Experimental Virology, Martinistraße 52, 20251 Hamburg, Germany;

[5]European XFEL GmbH, Holzkoppel 4, 22869 Schenefeld,

[6]University of Hamburg Medical Center Hamburg-Eppendorf, Martinistraße 52, 20246 Hamburg, Germany;

Present address:

[†]Norwich Medical School, Norwich Research Park, Colney Ln, Norwich, Norfolk, NR4 7UQ, United Kingdom;

[¿]Sosei Heptares, Steinmetz Building, Granta Park, Great Abington, Cambridge, CB21 6DG, United Kingdom;

[*] Corresponding author: eike.schulz@mpsd.mpg.de

Keywords:

Mtb, epoxide hydrolase A, crystal structure, α/β-hydrolase

Supplementary Information

Figure S1

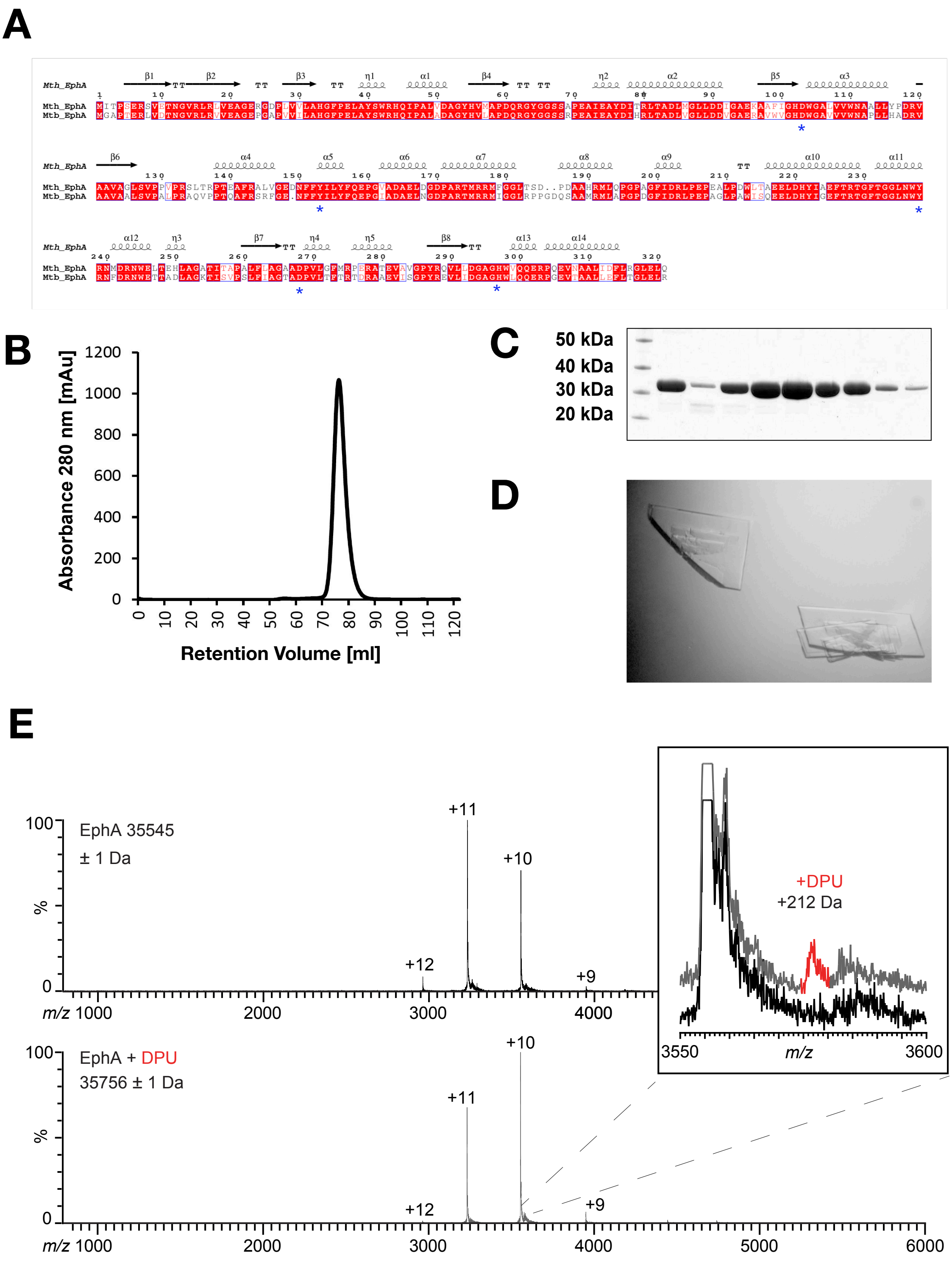

Figure S1: Mth-EphA/Mtb-EphA alignment, purification and crystallization **(A)** Mth-EphA is aligned to Mtb-EphA, secondary structure elements inferred from the Mth-EphA crystal structure are indicated. The alignment shows the high level of identity between both orthologs and demonstrates that Mth-EphA is a valid model for *M. tuberculosis* EphA, presumably exhibiting very similar structural characteristics. Catalytic active site residue are marked with *. (**B**) The Mth-EphA elution profile from size exclusion chromatography shows a sharp monodispersed peak reflecting the homogeneity of the sample. (**C**) A representative SDS-polyacrylamide gel from peak fractions of the purified protein shows clear, sharp bands demonstrating the purity of the sample. A molecular weight standard was loaded together with Mth-EphA. **(D)** Typical crystals obtained for Mth-EphA. The protein crystals often formed coadunate plates (right), however, occasionally individual, single crystals could be obtained (left). **(E)** Native mass spectra showing signals assigned to monomeric EphA (black) in 150 mM ammonium acetate, 1% butanediol, pH 7.5 (top) and additionally the protein-inhibitor complex (red) after incubation with 71 μM DPU and 0.2% DMSO (bottom). Magnification of the overlayed +10 peaks from both spectra displayed in the inset. Intensity of each +10 peak kept at 100%. An additional peak appears in presence of DPU corresponding to the complex (red).

Figure S2

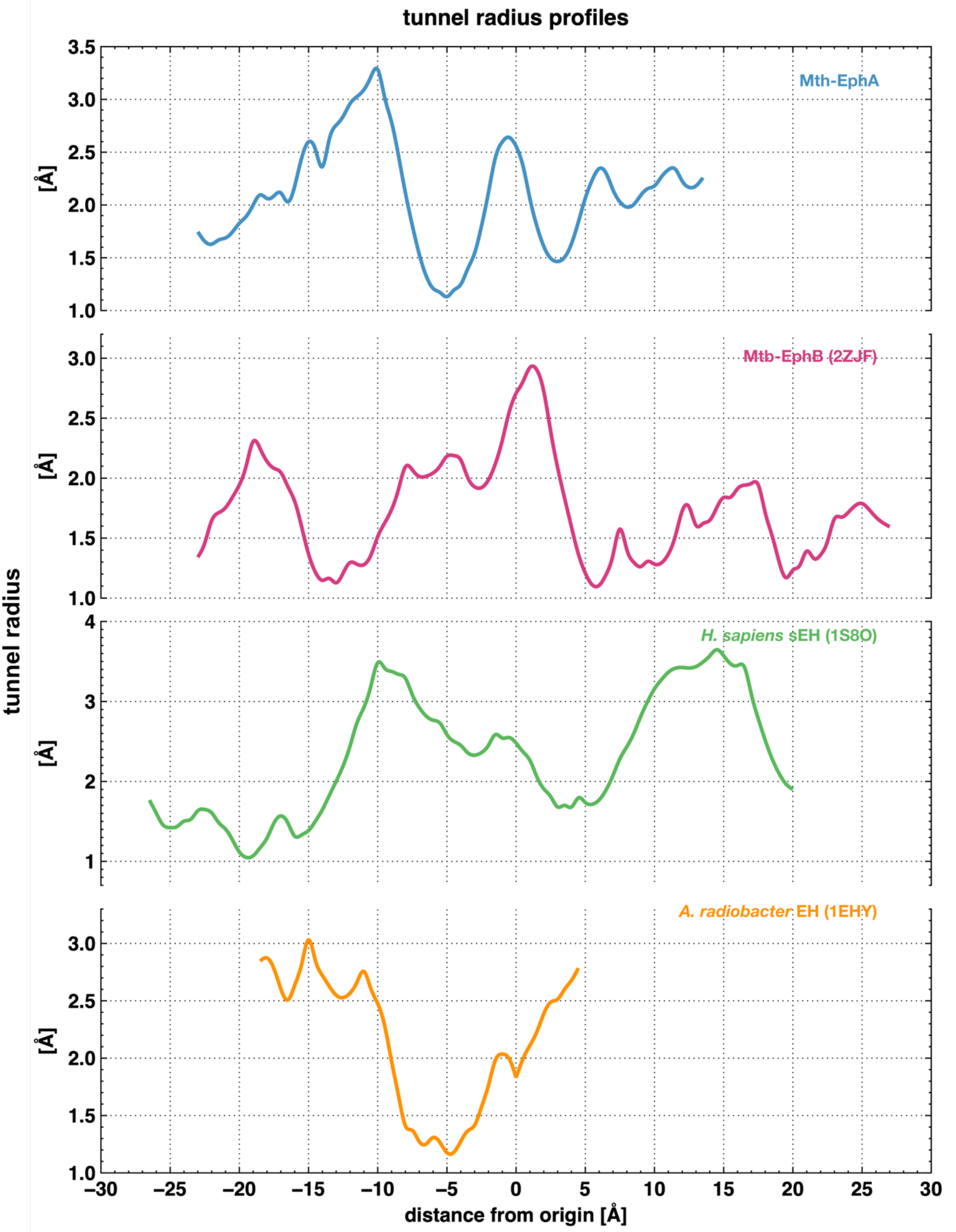

Figure S2: Active site tunnel radius profiles.

The active site tunnel radius (Å) is plotted against its distance from the origin in the active site (Å).

Figure S3

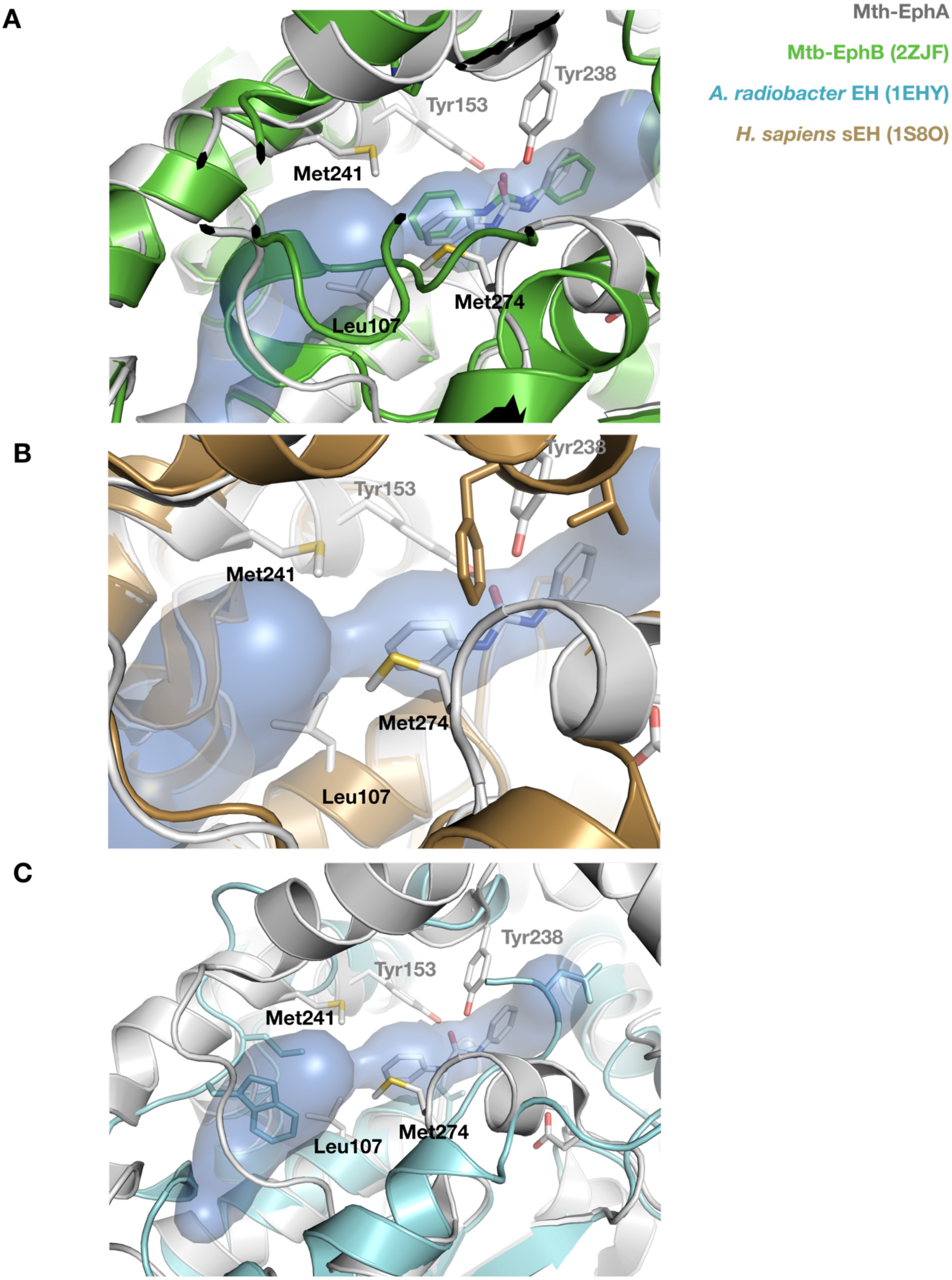

Figure S3: Superimposition of the Mth-EphA substrate

(**A**) Mth-EphA is shown in grey, (**B**) Mtb-EphB is shown in green, (**C**) sEH is shown in brown, *A. radiobacter* EH in cyan, the active site channel of Mth-EphA is depicted in blue. The superimposition

shows the partition of the Mth-EphA active site by Leu107, Met241 and Met274. Clearly the structures of the homologous EHs collide with the active site channel of Mth-EphA

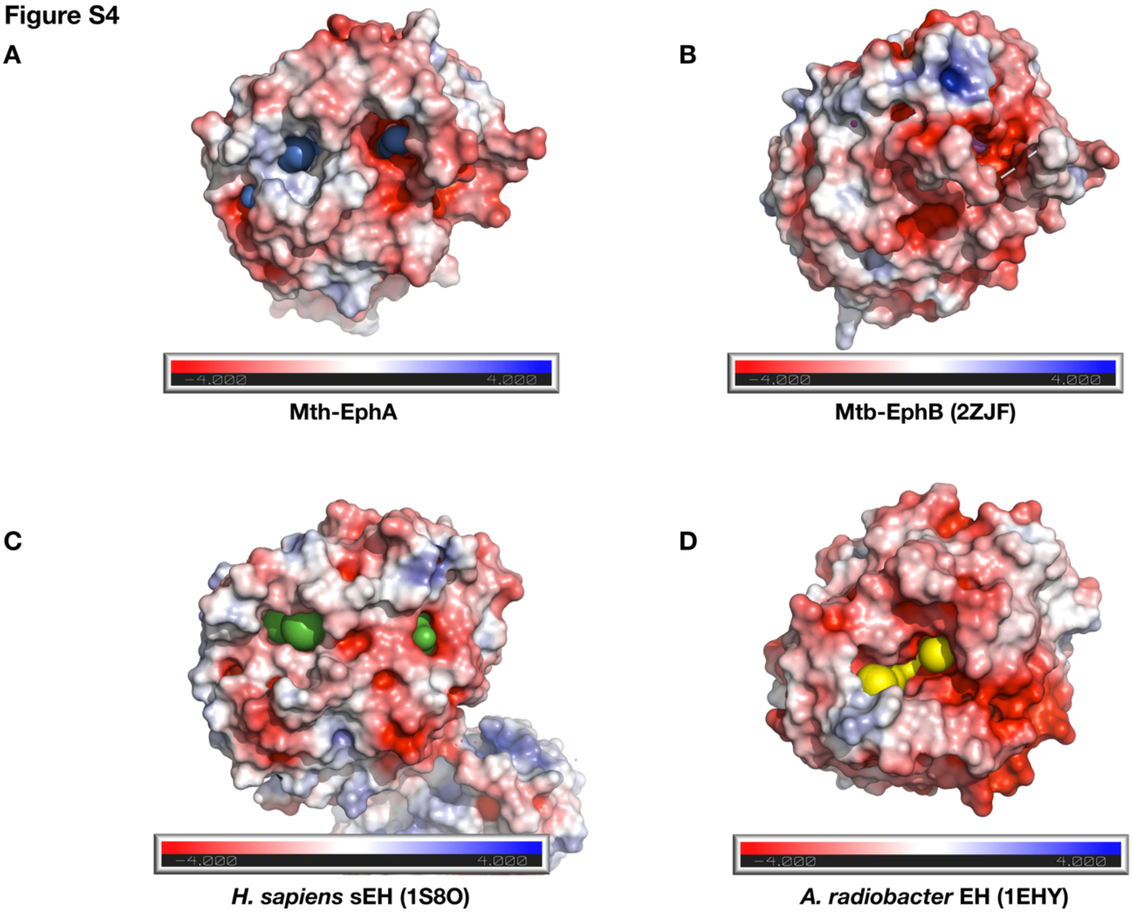

Figure S4: Surface charge representation of epoxide hydrolases

The APBS surface charge representation displays the different charge properties of the epoxide hydrolases. The active site tunnels are indicated according to the same color code as in Figure 4. Uniquely, Mth-EphA shows active site entry sites with opposing surface charges.

Figure S5

A

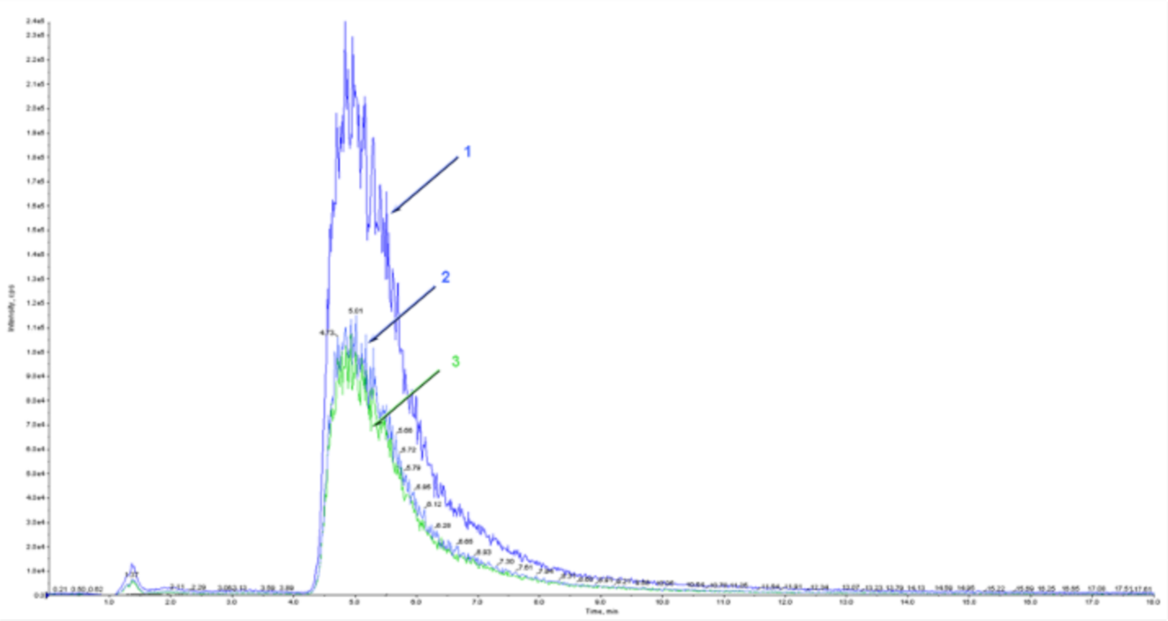

B

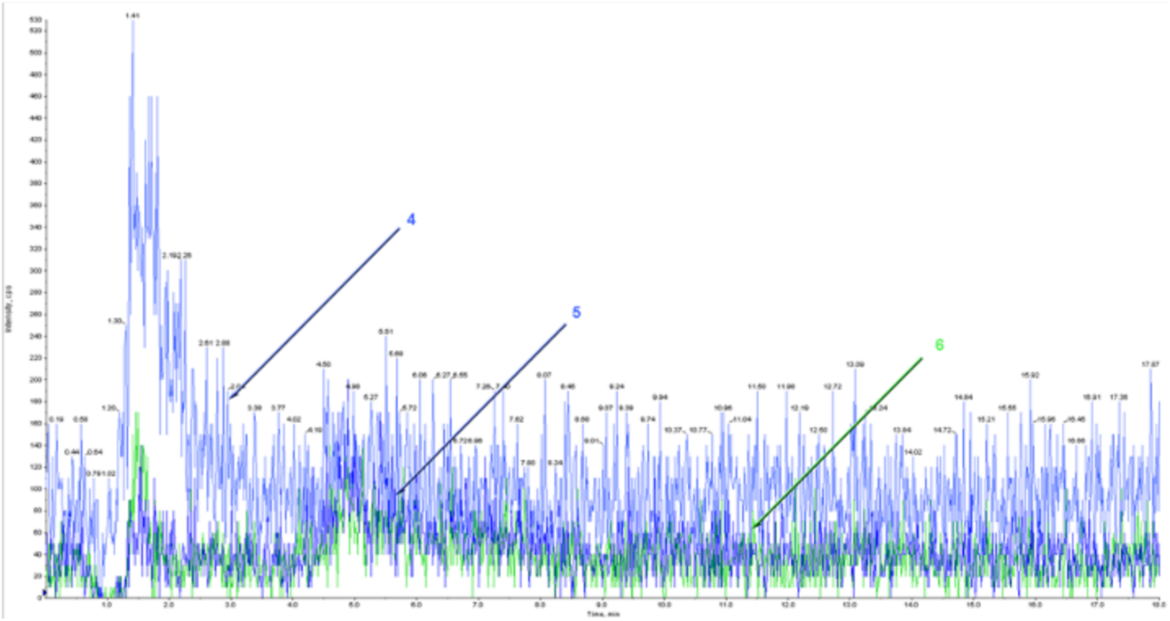

Fig. S5 MRM chromatograms of cis-stilben oxide

(**A**) MRM chromatogram of cis-stilben oxide after treatment with Mth-EphA (assay sample). Parent ion m/z: 196.9; fragment ion m/z: 105 (1), 77 (2), 91 (3).

(**B**). Assignment of three MRM chromatogram traces from Fig. S5a. Parent ion m/z: 215; fragment ion m/z: 215 (4), 105 (5), 77 (6).

Figure S6

A

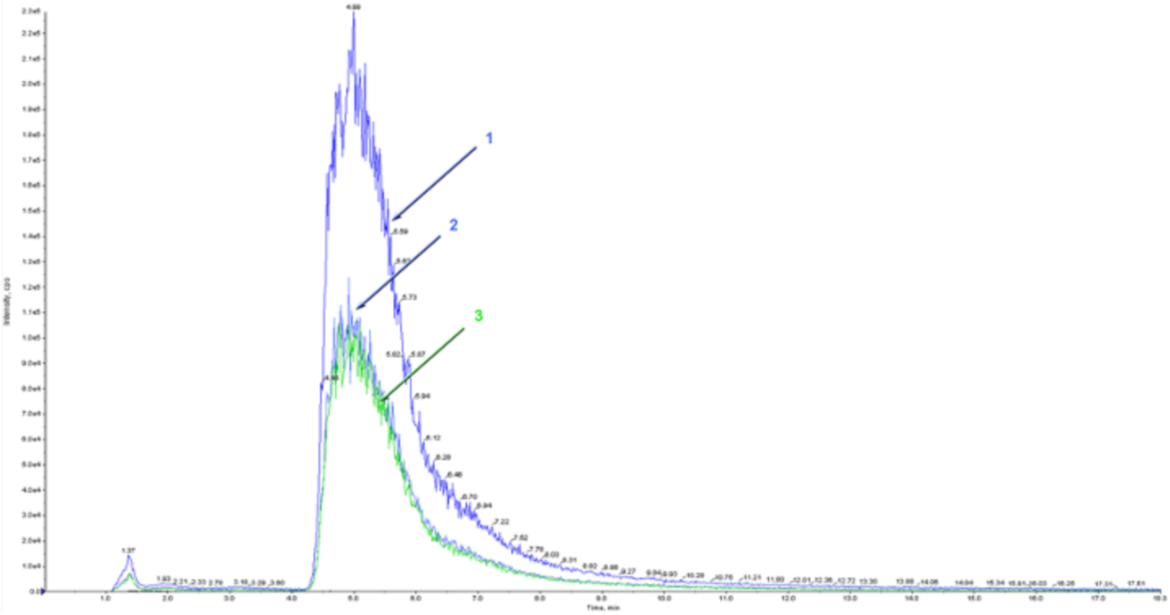

B

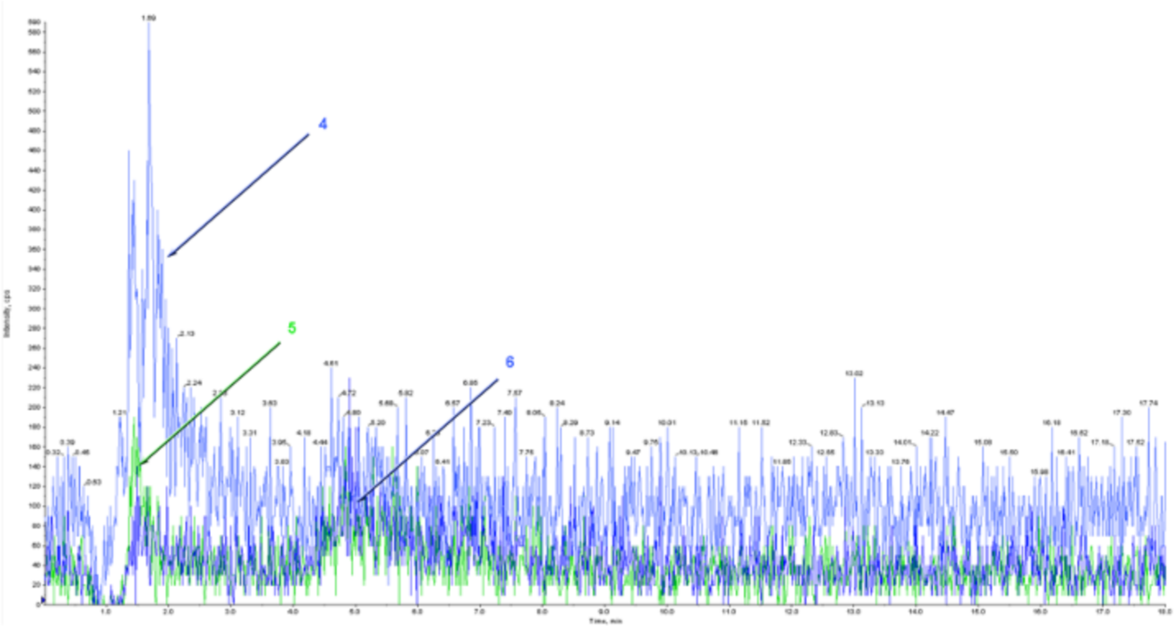

Fig. S6 MRM chromatogram of trans-stilben oxide

(A) MRM chromatogram of trans-stilben oxide after treatment with Mth-EphA (assay sample). Parent ion m/z: 196.9, fragment ion m/z: 105 (1), 77 (2), 91 (3).

(B) Assignment of three MRM chromatogram traces from Fig. S6a. Parent ion m/z: 215; fragment ion m/z: 215 (1), 77 (2), 105 (3).

Figure S7

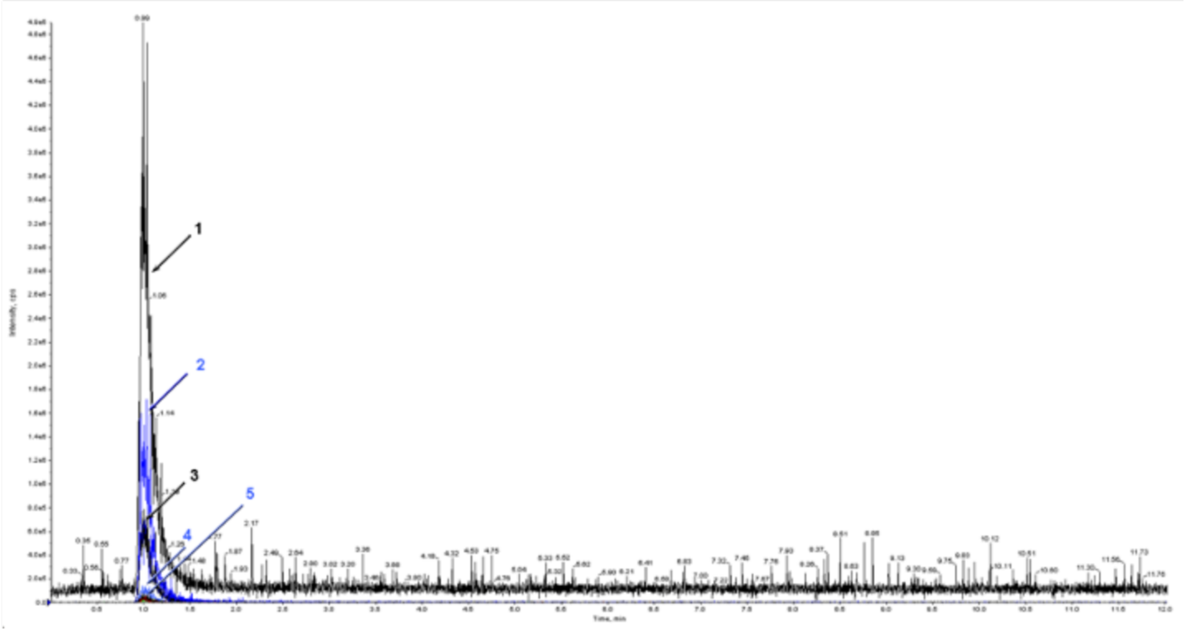

Fig. S7. MRM chromatogram of the standard compound hydrobenzoin.

Parent ion m/z: 215; fragment ion m/z: 215 (1), 105 (2), 91 (3), 77 (4), 51 (5).

Figure S8

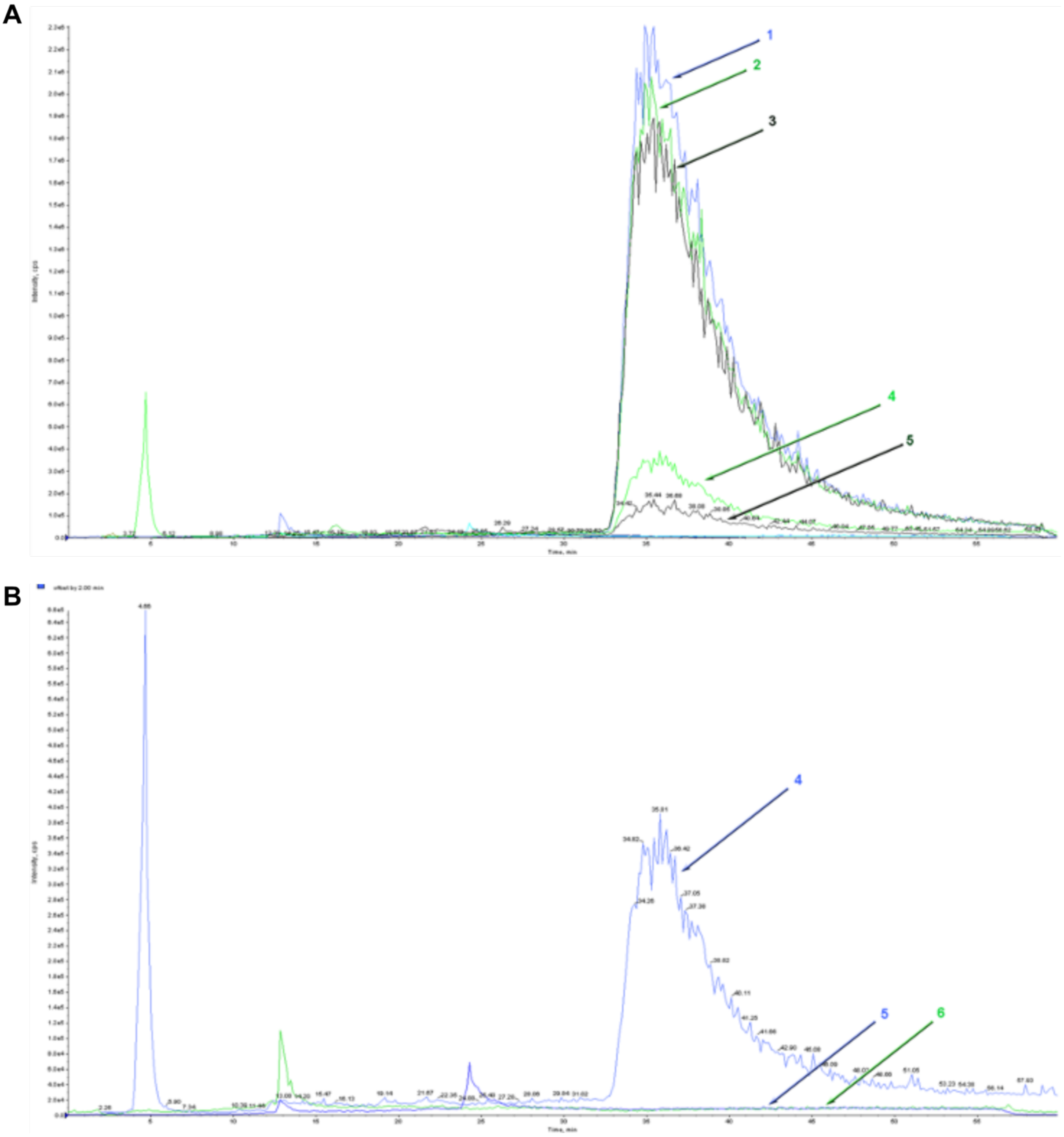

Fig. S8 MRM chromatogram of trans-1,3-diphenyl-2,3-epoxypropan-1-one – control reaction without enzyme.

(A) MRM chromatogram of trans-1,3-diphenyl-2,3-epoxypropan-1-one incubated in reaction buffer *without Mth-EphA* (control sample). Parent ion m/z → fragment ion m/z: 225 → 105 (1), 225 → 225 (2), 242 → 242 (3), 243 → 243 (4), 247 → 247 (5).

(B) Assignment of three MRM chromatogram traces from Fig. S8a. Parent ion m/z → fragment ion m/z: 243 → 243 (4), 265 → 265 (5), 260 → 260 (6).

Figure S9

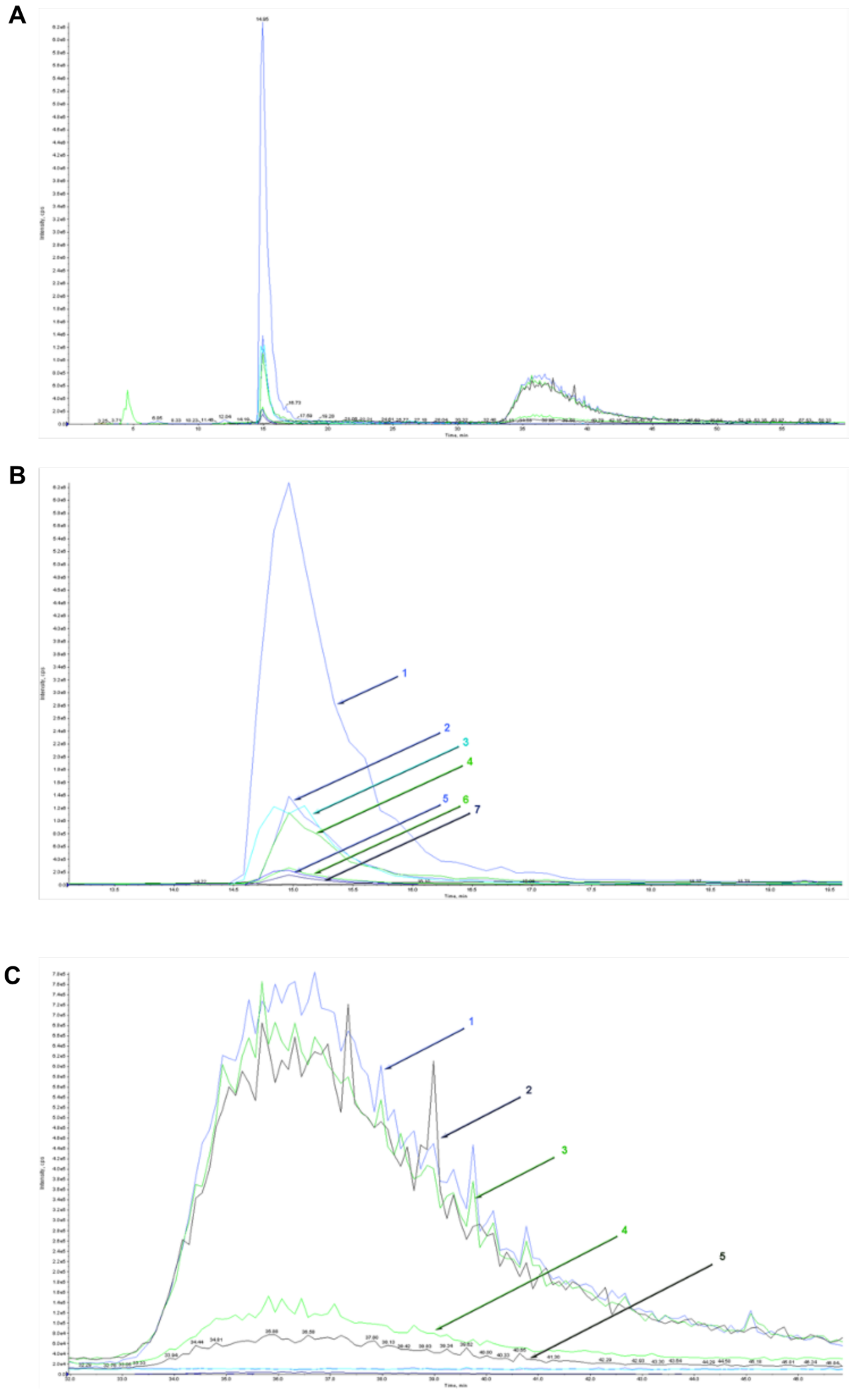

Fig. S9 MRM chromatogram of trans-1,3-diphenyl-2,3-epoxypropan-1-one

(A) MRM chromatogram of trans-1,3-diphenyl-2,3-epoxypropan-1-one treated with Mth-EphA (assay sample). For chromatogram trace assignment see the figures S5b and S5c.

(B) Enlarged section I of the MRM chromatogram (Fig. S9a) of trans-1,3-diphenyl-2,3-epoxypropan-1-one treated with Mth-EphA (assay sample). Parent ion m/z → fragment ion m/z: 260 → 260 (1), 224.8 → 105 (2), 265 → 265 (3), 224.8 → 224.8 (4), 260 → 225 (5), 243 → 243 (6), 243 → 225 (7).

(C) Enlarged section II of the MRM chromatogram (Fig. S9a) of trans-1,3-diphenyl-2,3-epoxypropan-1-one treated with Mth-EphA (assay sample). Parent ion m/z → fragment ion m/z: 224.8 → 105 (1), 242 → 242 (2), 224.8 → 224.8 (3), 243 → 243 (4), 247 → 247 (5).

Figure S10

A

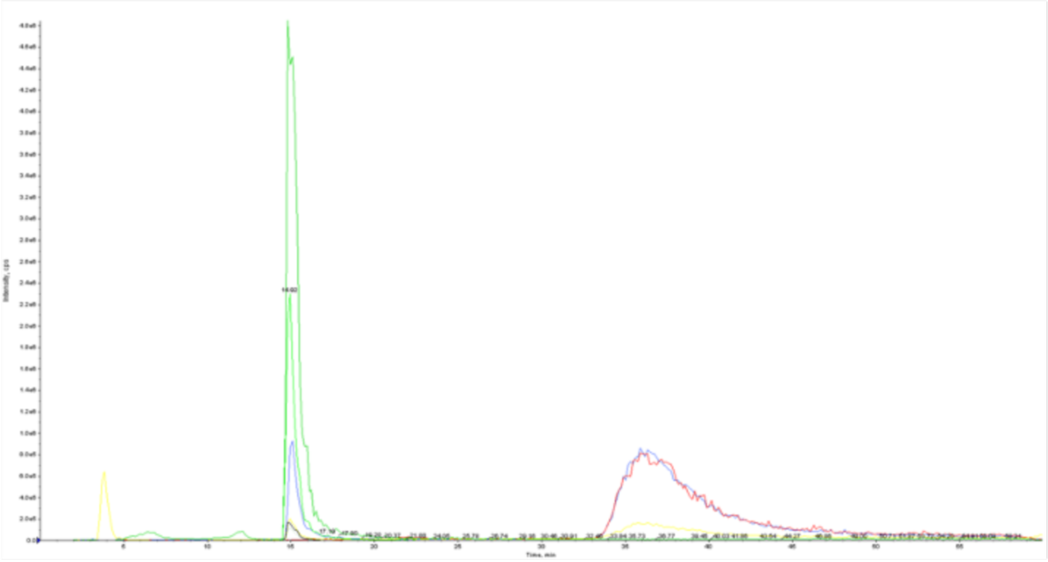

B

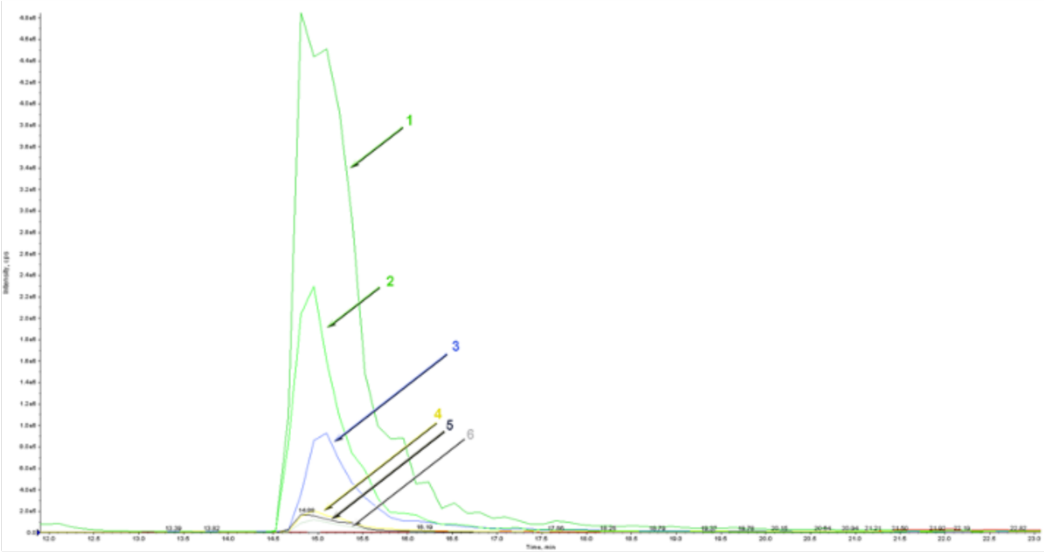

C

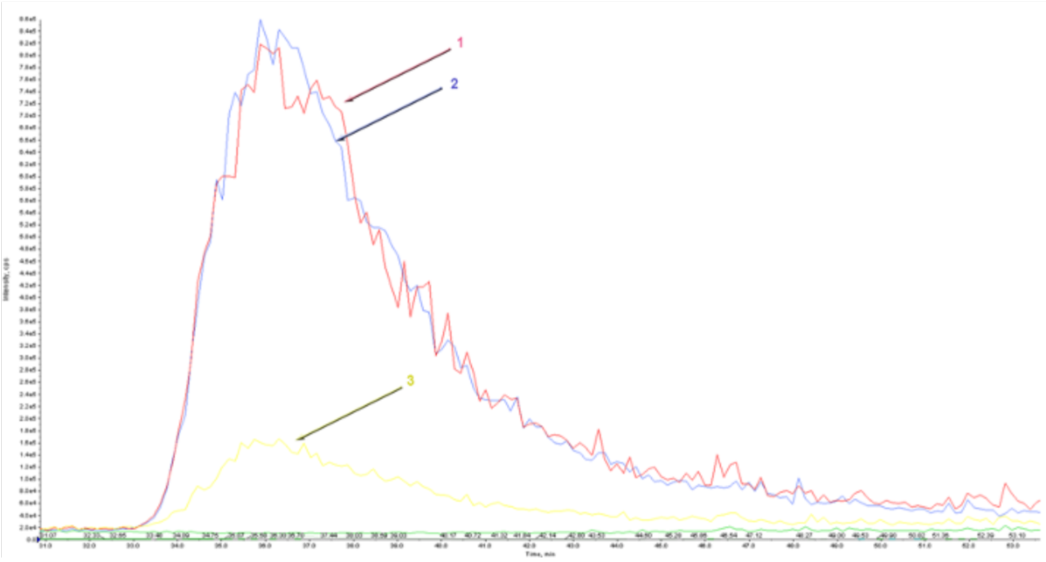

Fig. S10 MRM chromatogram of trans-1,3-diphenyl-2,3-epoxypropan-1-one

(A) MRM chromatogram of trans-1,3-diphenyl-2,3-epoxypropan-1-one treated with Mth-EphA (assay sample). For chromatogram trace assignment see the figures S6b and S6c.

(B) Enlarged section I of the MRM chromatogram (Fig. S10a) of trans-1,3-diphenyl-2,3-epoxypropan-1-one treated with Mth-EphA (assay sample). Parent ion m/z → fragment ion m/z: 260 → 260 (1), 260 → 119 (2), 224.8 → 224.8 (3), 243 → 243 (4), 260 → 225 (5), 243 → 225 (6).

(C) Enlarged section II of the MRM chromatogram (Fig. S10a) of trans-1,3-diphenyl-2,3-epoxypropan-1-one treated with Mth-EphA (assay sample). Parent ion m/z → fragment ion m/z: 242 → 242 (1), 224.8 → 224.8 (2), 243 → 243 (3).

Figure S11

A

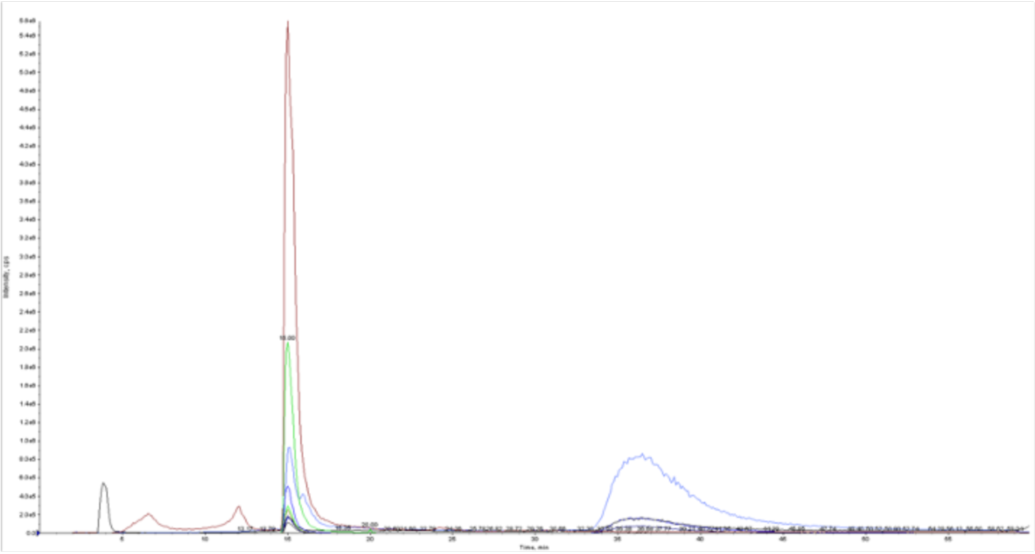

B

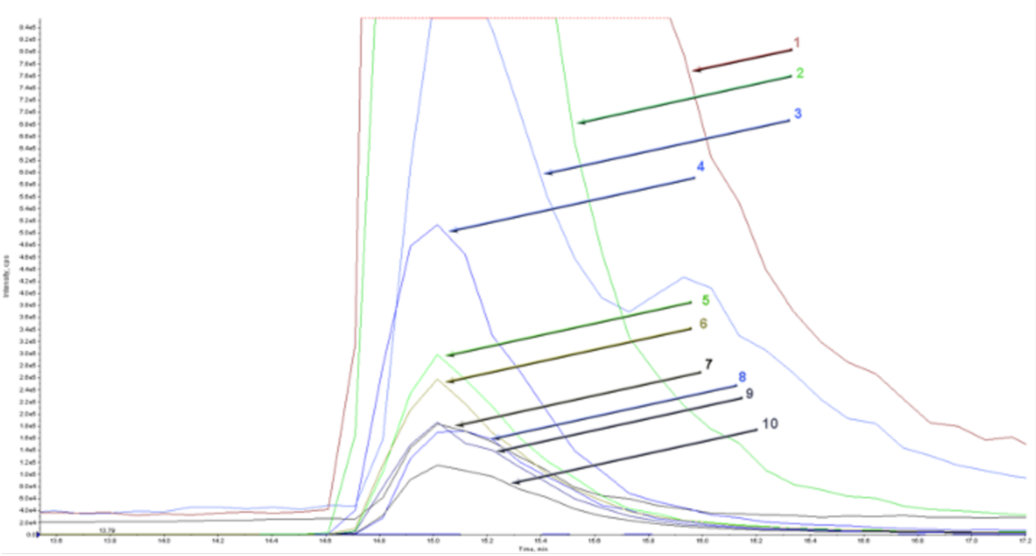

C

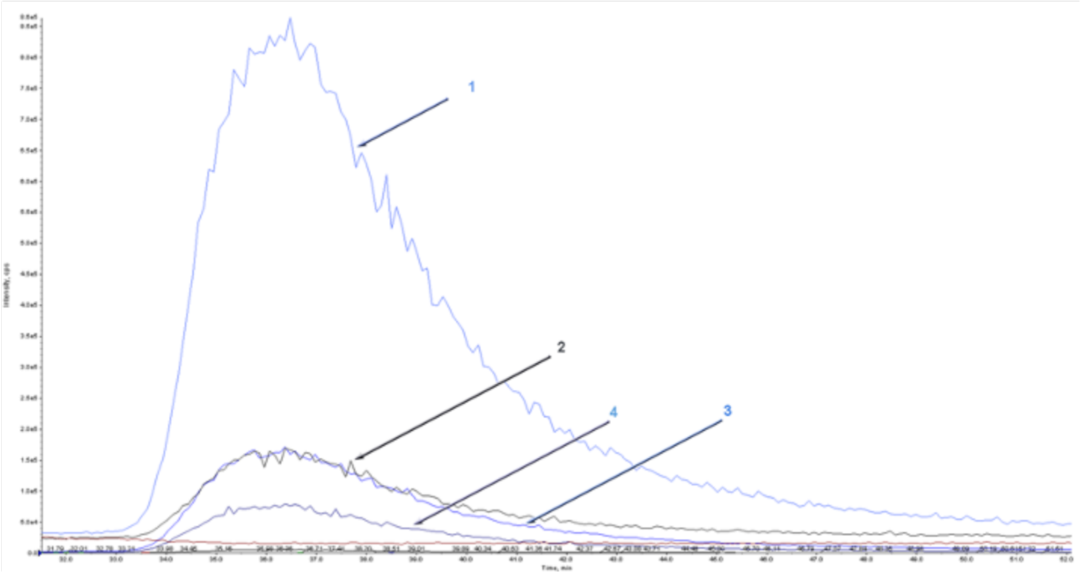

Fig. S11 MRM chromatogram of trans-1,3-diphenyl-2,3-epoxypropan-1-one

(A) MRM chromatogram of trans-1,3-diphenyl-2,3-epoxypropan-1-one treated with Mth-EphA (assay sample). For chromatogram trace assignment see the figures S7b and S7c.

(B) Enlarged section I of the MRM chromatogram (Fig. S11a) of trans-1,3-diphenyl-2,3-epoxypropan-1-one treated with Mth-EphA (assay sample). Parent ion m/z → fragment ion m/z: 260 → 260 (1), 260 → 105 (2), 224.8 → 224.8 (3), 260 → 119 (4), 243 → 119 (5), 260 → 225 (6) , 243 → 243 (7) , 243 → 105 (8) , 224.8 → 105 (9) , 243 → 225 (10).

(C) Enlarged section II of the MRM chromatogram (Fig. S11a) of trans-1,3-diphenyl-2,3-epoxypropan-1-one treated with Mth-EphA (assay sample). Parent ion m/z → fragment ion m/z: 224.8 → 224.8 (1), 243 → 243 (2), 224.8 → 105 (3), 243 → 105 (4).
